# Machine Learning on Toxicogenomic Data Reveals a Strong Association Between the Induction of Drug-Metabolizing Enzymes and Centrilobular Hepatocyte Hypertrophy in Rats

**DOI:** 10.3390/ijms26104886

**Published:** 2025-05-20

**Authors:** Kazuki Ikoma, Takuomi Hosaka, Akira Ooka, Ryota Shizu, Kouichi Yoshinari

**Affiliations:** Laboratory of Molecular Toxicology, School of Pharmaceutical Sciences, University of Shizuoka, 52-1 Yada, Suruga-ku, Shizuoka 422-8526, Japanhosaka@u-shizuoka-ken.ac.jp (T.H.); ohoka@u-shizuoka-ken.ac.jp (A.O.); r_shizu@u-shizuoka-ken.ac.jp (R.S.)

**Keywords:** enzyme induction, hepatocyte hypertrophy, machine learning, toxicity mechanism

## Abstract

Centrilobular hepatocyte hypertrophy is frequently observed in animal studies for chemical safety assessment. Although its toxicological significance and precise mechanism remain unknown, it is considered an adaptive response resulting from the induction of drug-metabolizing enzymes (DMEs). This study aimed to elucidate the association between centrilobular hepatocyte hypertrophy and DME induction using machine learning on toxicogenomic data. Utilizing publicly available gene expression data and pathological findings from rat livers of 134 compounds, we developed six different types of machine learning models to predict the occurrence of centrilobular hepatocyte hypertrophy based on gene expression data as explanatory variables. Among these, a LightGBM-based model demonstrated the best performance with an accuracy of approximately 0.9. With this model, we assessed each gene’s contribution to predicting centrilobular hepatocyte hypertrophy using mean absolute SHAP values. The results revealed that *Cyp2b1* had an extremely significant contribution, while other DME genes also displayed positive contributions. Additionally, enrichment analysis of the top 100 genes based on mean absolute SHAP values identified “Metabolism of xenobiotics by cytochrome P450” as the most significantly enriched term. In conclusion, the current results suggest that the induction of multiple DMEs, including CYP2B1, is crucial for the development of centrilobular hepatocyte hypertrophy.

## 1. Introduction

Hepatomegaly and hepatocyte hypertrophy are commonly observed effects of administration of chemical substances in toxicological studies involving experimental animals [1,2,3]. Hepatocyte hypertrophy can occur in specific liver regions, particularly in the centrilobular region. For example, phenobarbital, an antiepileptic drug, induces centrilobular hepatocyte hypertrophy in mice and rats, accompanied by the induction of microsomal drug-metabolizing enzymes (DMEs), including cytochrome P450s (P450s), along with the proliferation of smooth endoplasmic reticulum [2,3,4,5,6]. The underlying mechanism of centrilobular hepatocyte hypertrophy remains poorly understood. However, since many P450 inducers, such as phenobarbital, cause hepatocyte hypertrophy specifically in the centrilobular region, where they primarily induce DMEs, it is hypothesized that centrilobular hepatocyte hypertrophy occurs as a secondary effect of DME induction [2,3,4,5,6]. Nevertheless, there is no direct evidence to suggest that DME induction directly causes centrilobular hepatocyte hypertrophy.

In chemical toxicity studies, hepatocyte hypertrophy associated with DME induction is regarded as an adaptive change rather than a toxic effect, unless hepatic necrosis or elevated liver injury markers, such as alanine aminotransferase, γ-glutamyl transpeptidase, and alkaline phosphatase, are present [2,5]. However, a relationship between hepatocyte hypertrophy and hepatocarcinogenesis has been suggested [7,8,9]. For example, long-term administration of WY-14643, an agonist for peroxisome proliferator-activated receptor alpha (PPARα), or phenobarbital, which indirectly activates the nuclear receptor constitutive androstane receptor (CAR) through phosphorylation/dephosphorylation signaling [10], leads to hepatocyte hypertrophy, hepatocyte proliferation, and subsequently, liver cancer in rodents [11,12,13,14,15]. Furthermore, kava root extract, derived from a plant in the Piperaceae family, has been reported to induce both hepatocyte hypertrophy and elevated serum γ-glutamyl transpeptidase and alkaline phosphatase levels in rats [4]. These findings suggest that hepatocyte hypertrophy induced by certain chemical compounds is linked to hepatotoxicity, necessitating further exploration of its mechanisms and toxicological significance.

Hepatocyte hypertrophy is also observed and plays a role in liver regeneration. Using mouse partial hepatectomy models, Miyaoka et al. [16] demonstrated that liver regeneration after 30% hepatectomy is completed by hepatocyte hypertrophy without cell proliferation, and after 70% hepatectomy, both proliferation and hypertrophy contribute to regeneration. More recently, another group demonstrated that hypertrophy of mid-lobular hepatocytes largely contributes to liver regeneration after CCl_4_-induced liver injury and two-thirds partial hepatectomy in mice [17]. Since the expression of DMEs, including P450s, is generally decreased during liver regeneration [18,19], the mechanisms may differ between hepatocyte hypertrophy induced by chemical exposure and that observed during liver regeneration.

Open TG-GATEs (Toxicogenomics Project-Genomics Assisted Toxicity Evaluation System) [20] is a comprehensive toxicogenomics database containing gene expression data from livers and kidneys, as well as toxicity evaluation data, including hepatic and renal pathological findings and blood biochemistry results, from rats treated with over 100 chemical substances across multiple doses and durations. These data are publicly available through the Toxygates web platform [21]. Nagata et al. [22] used Open TG-GATEs data to explore genes contributing to increased relative liver weight, while Liu et al. [9] identified characteristic genes associated with hepatocyte hypertrophy leading to hepatocarcinogenesis.

Machine learning methods such as LightGBM [23] and XGBoost [24] have been developed and applied across various fields for prediction and classification tasks, achieving higher accuracy than conventional approaches. However, their predictive rationale often remains a black box. To address this limitation, SHapley Additive exPlanations (SHAP) [25] has been proposed as an explainable artificial intelligence (XAI) method that calculates the contribution of each feature to predictions based on game theory. In chemical toxicity prediction, SHAP has been utilized to identify critical structural features associated with toxicity [26] and to explore toxicity biomarkers [27].

In the present study, we aimed to elucidate the mechanisms of hepatocyte hypertrophy using machine learning approaches. Specifically, we developed prediction models for chemical-induced centrilobular hepatocyte hypertrophy based on gene expression data from rat studies in Open TG-GATEs. Furthermore, we analyzed the model’s predictive rationale using SHAP to quantitatively evaluate the contribution of each gene to the prediction and to identify genes that are crucial for the development of centrilobular hepatocyte hypertrophy.

## 2. Results

### 2.1. Dataset

We constructed a dataset by merging data from two sources. First, we collected liver pathological findings from rat repeated-dose toxicity studies in Open TG-GATEs, which included 134 compounds administered at three doses (low, medium and high) over four different durations (3, 7, 14, or 28 days). We then combined this with the corresponding gene expression data from individual rats, obtained from Toxygates by using the sample ID as a matching key. The resulting dataset comprised dosing results from a total of 5055 rats.

In this dataset, the positive rate for centrilobular hepatocyte hypertrophy across all samples was approximately 10%, indicating an imbalanced dataset with relatively few positive cases (Table 1). However, this positive rate increased with higher doses and longer administration periods, showing considerable variation, with rates ranging from 0% to 28.0% among the 12 groups stratified by dose and administration period (Table 1).

The distribution of samples across the five folds was well balanced (Table 2), with each fold containing between 991 and 1039 administration results (samples) and between 28 and 30 unique compounds. The positive rates were also relatively consistent across the folds, ranging from 8.0% to 11.1%, which is close to the overall positive rate of 10.1% for the complete dataset. This balanced distribution of both samples and positive cases across the folds helped ensure the reliability of our cross-validation results by minimizing potential bias from data splitting.

Supplementary Table S1 provides a comprehensive overview of the dataset, including compound names, administration durations, dose levels, labels (e.g., presence or absence of centrilobular hepatocyte hypertrophy), and assignments to the five folds for cross-validation.

### 2.2. Training and Validation of Prediction Models for Centrilobular Hepatocyte Hypertrophy

Using the constructed dataset, we trained and validated six different machine learning models: logistic regression, support vector machines (SVM), random forest (RF), XGBoost, LightGBM, and *k*-nearest neighbors (kNN). The presence or absence of centrilobular hepatocyte hypertrophy served as the objective variable, while gene expression ratios (log2 fold changes) relative to the vehicle control group were used as explanatory variables. We performed stratified five-fold cross-validation, dividing the 134 compounds into five groups to minimize the differences in positive rates among the groups (Supplementary Table S2).

**Figure 1:**
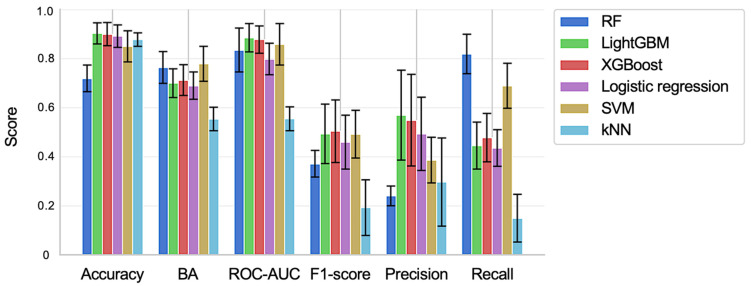
Performance of the machine learning models. The evaluation results for each prediction model are shown, with error bars representing the standard deviation of each metric.

Figure 1 presents the evaluation results for each prediction model. Both LightGBM and XGBoost, which are gradient-boosting algorithms, demonstrated superior performance across various accuracy metrics compared to the other models. Specifically, LightGBM achieved the highest area under the receiver operating characteristic curve (ROC-AUC). After implementing early stopping, the mean ROC-AUC for LightGBM slightly improved from 0.885 to 0.887. The SVM exhibited the highest balanced accuracy (BA) of 0.78, while the RF showed a notably high recall of 0.82. However, it is important to note that BA and recall require the binarization of prediction results, and these evaluation metrics can vary significantly depending on the selected binarization threshold. Additionally, precision and F1-score exhibited greater variability across different splits in the five-fold cross-validation than the other evaluation metrics for all models.

### 2.3. Genes Contributing to the Prediction of Centrilobular Hepatocyte Hypertrophy

To identify the genes most influential in predicting centrilobular hepatocyte hypertrophy, we focused on the LightGBM model, which achieved the highest ROC-AUC score. We evaluated each gene’s average contribution to the model’s predictions using mean absolute SHAP values. Out of the 14,037 genes analyzed, 4946 had mean absolute SHAP values greater than 0, indicating that approximately one-third of the genes were involved in the prediction process. The names and mean absolute SHAP values of these contributing genes are provided in Supplementary Table S3. Notably, the gene with the most significant contribution to the predictions was *Cyp2b1*, a CAR-target rat P450 gene, with a mean absolute SHAP value considerably higher than that of other genes.

**Figure 2:**
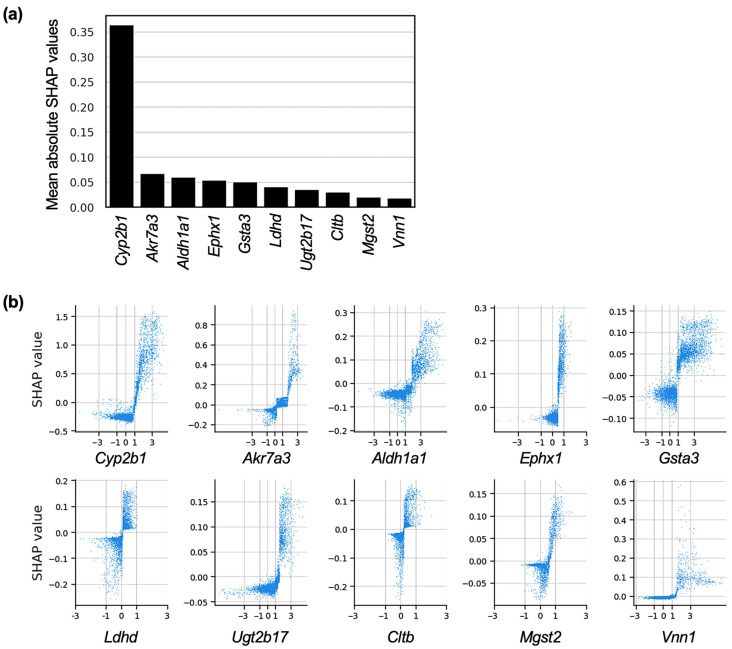
SHAP analysis of genes’ contributions to model prediction. (**a**) A bar graph of the top 10 genes with mean absolute SHAP values is shown. (**b**) Relationships between SHAP values (*y*-axis) and Log2FC (*x*-axis) for the top 10 genes for mean absolute SHAP values are shown as scatter plots.

Several of the top 10 genes, ranked by mean absolute SHAP values, were DME genes, including *Akr7a3* and *Aldh1a1* (Figure 2a). These top 10 genes showed a positive correlation between their fold-change values on a logarithmic scale with base 2 (Log2(fold-change); Log2FC) and SHAP values (Figure 2b). These findings suggest that the upregulation of DMEs, particularly CYP2B1, significantly contributes to the development of centrilobular hepatocyte hypertrophy.

### 2.4. Enrichment Analysis Using the Top 100 Genes That Contributed to the Prediction

**Figure 3:**
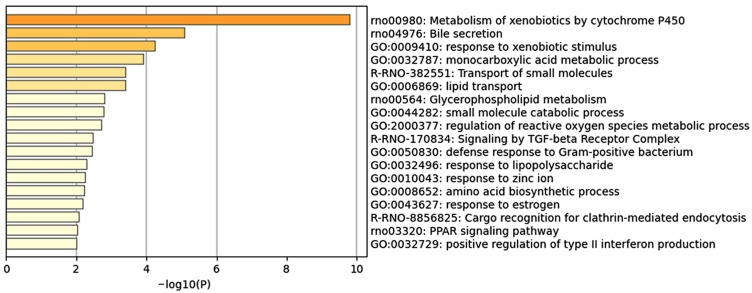
Enrichment analysis using the top 100 genes for mean absolute SHAP values. A bar graph of the enriched terms is shown.

To gain biological insights into the genes that significantly contribute to predicting centrilobular hepatocyte hypertrophy, we conducted an enrichment analysis using the top 100 genes ranked by mean absolute SHAP values from the LightGBM model (Figure 3). The most significantly enriched term was “Metabolism of xenobiotics by cytochrome P450”, which included five of the top ten genes: *Cyp2b1*, *Akr7a3*, *Ephx1*, *Gsta3*, and *Ugt2b17*. This finding strongly suggests that the induction of DMEs, including P450 enzymes, plays a central role in the development of centrilobular hepatocyte hypertrophy.

In addition to the P450-related term, several other enriched terms were associated with drug exposure and metabolism, such as “Response to xenobiotic stimulus” and “Monocarboxylic acid metabolic process”. These findings support the idea that the induction of DMEs, particularly P450s, is a crucial factor in the pathogenesis of centrilobular hepatocyte hypertrophy.

### 2.5. Case Studies of Samples Showing CYP2B1 Induction with or Without Centrilobular Hepatocyte Hypertrophy

To further investigate the relationship between CYP2B1 induction and centrilobular hepatocyte hypertrophy, we analyzed individual samples, including those with CYP2B1 induction, with or without centrilobular hepatocyte hypertrophy. For example, samples treated with the typical enzyme inducer phenobarbital [6,12] at high doses for 28 days (Phenobarbital/High/28-day) showed a *Cyp2b1* Log2FC value of 3.828 and exhibited centrilobular hepatocyte hypertrophy. In contrast, samples treated with nifedipine at high doses for 28 days (Nifedipine/High/28-day) demonstrated a similar *Cyp2b1* Log2FC value of 3.710 but did not exhibit centrilobular hepatocyte hypertrophy. Notably, nifedipine administration did not induce centrilobular hepatocyte hypertrophy at any dose level or duration (Supplementary Table S1).

**Figure 4:**
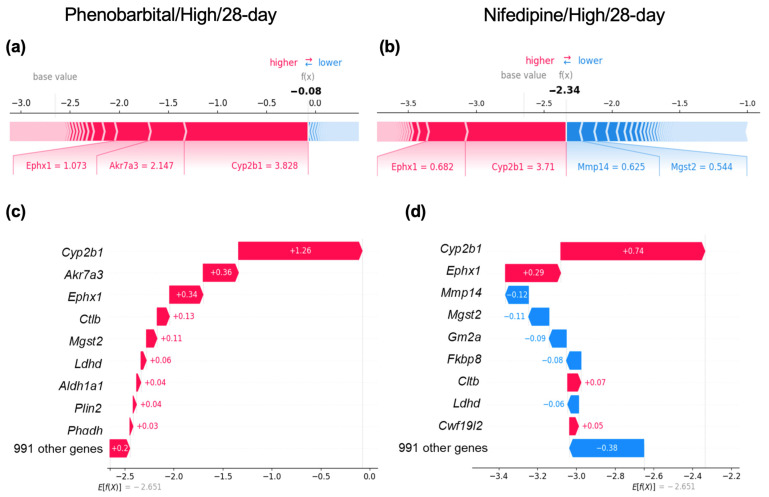
Case studies of SHAP analyses on gene contributions to model predictions. (**a**,**b**): SHAP value plots illustrating the impact of individual genes on model predictions for Phenobarbital/high/28-day (high-dose phenobarbital administration for 28 days) and Nifedipine/high/28-day (high-dose nifedipine administration for 28 days). Red bars indicate genes that positively contribute to the predictions, while blue bars represent genes that show negative contributions. The values shown are the log2FC of gene expression. (**c**,**d**): Waterfall plots visualizing the cumulative impact of genes on model predictions. The x-axis represents SHAP values, indicating the magnitude of each gene’s contribution. The value presented is the SHAP value of the gene.

To investigate why nifedipine did not induce centrilobular hepatocyte hypertrophy despite inducing *Cyp2b1* to levels comparable to phenobarbital, we quantified the contribution of each gene to the prediction using SHAP value analysis for these two specific treatment conditions. The SHAP value analysis for Phenobarbital/High/28-day revealed positive values not only for *Cyp2b1* but also for other DME genes, including *Akr7a3*, *Ephx1*, and *Mgst2* (Figure 4a,c), indicating that these DMEs positively contributed to the prediction of centrilobular hepatocyte hypertrophy in the LightGBM model. In contrast, the SHAP value analysis for Nifedipine/High/28-day indicated that although *Cyp2b1* and *Ephx1* had positive values signifying positive contributions to the prediction, these values were smaller than those in Phenobarbital/High/28-day, suggesting a lesser degree of contribution (Figure 4b,d). Additionally, *Akr7a3*, which showed significant positive contributions in Phenobarbital/High/28-day, demonstrated minimal contribution in Nifedipine/High/28-day, while *Mgst2*, which contributed positively in Phenobarbital/High/28-day, exhibited a negative contribution in Nifedipine/High/28-day (Figure 4b,d).

This pattern of significant *Cyp2b1* contribution, along with limited involvement from other DME genes, was also observed with chemicals such as disopyramide and clomipramine, which induce CYP2B1 without leading to centrilobular hepatocyte hypertrophy. The Log2FC values for *Cyp2b1* in high-dose, 28-day samples were 3.29 for disopyramide and 3.63 for clomipramine (Supplementary Figure S1).

These results suggest that although the induction of CYP2B1 significantly contributes to the development of centrilobular hepatocyte hypertrophy, it is insufficient on its own; multiple DMEs must also be induced for this to occur.

## 3. Discussion

In our LightGBM model for predicting centrilobular hepatocyte hypertrophy, *Cyp2b1* exhibited the most significant contribution to the prediction (Figure 2a). Other DME genes, including *Aldh1a1* and *Akr7a3*, also demonstrated substantial contributions, as samples with higher induction ratios of these DMEs tended to contribute more strongly to the prediction (Figure 2b). Furthermore, enrichment analysis of the top 100 genes with the highest contributions revealed that several DME-related terms were enriched (Figure 3). These findings suggest a close involvement of DME induction in the development of centrilobular hepatocyte hypertrophy. Chemical-induced CYP2B/3A induction is primarily observed in the centrilobular region, and many of these enzyme-inducing chemicals cause centrilobular hepatocyte hypertrophy [6]. Given that enzyme induction and hepatocyte hypertrophy are observed in the same region of the liver lobule, centrilobular hepatocyte hypertrophy is regarded as a secondary effect resulting from DME induction [2,3,4,5,6]. Our machine learning results using gene expression data support this hypothesis.

CYP2B induction by phenobarbital, a typical enzyme-inducing drug, is mediated through the activation of the nuclear receptor CAR [12,28,29]. Therefore, centrilobular hepatocyte hypertrophy can occur via different signals associated with CAR activation, beyond those involved in enzyme induction. While CAR activation promotes hepatocyte proliferation in rodents [11,12,13,14,30,31], the enrichment analysis did not reveal terms related to cell proliferation (Figure 3). These results suggest that the development of centrilobular hepatocyte hypertrophy is strongly linked to DME induction but has a limited association with other CAR functions, supporting the idea that, at least for CAR activators, centrilobular hepatocyte hypertrophy is a secondary phenomenon associated with DME induction resulting from CAR activation, rather than the diverse signals resulting from CAR activation.

While our dataset included 44 P450 genes from the drug-metabolizing CYP1-CYP4 subfamilies, only *Cyp2b1* significantly contributed to the prediction of centrilobular hepatocyte hypertrophy. The induction of P450 involves xenobiotic-responsive transcription factors such as CAR, PXR, and AHR, with PXR primarily involved in CYP3A induction, CAR in CYP2B induction, and AHR in CYP1A induction [6,32,33]. Furthermore, the crosstalk between PXR and CAR in cooperatively inducing target gene expression is well-documented [34]. Previous studies have shown that many activators of these transcription factors induce centrilobular hepatocyte hypertrophy, suggesting that multiple P450 forms could contribute to predicting this condition. However, the contribution of P450 to the prediction was almost exclusively limited to CYP2B1.

When examining the median Log2FC values for each P450 gene across all the samples, *Cyp2b1* showed the highest value, significantly exceeding the median values of other P450 forms (Supplementary Table S4). These results suggest that among P450s, CYP2B1 induction may be particularly important for the development of centrilobular hepatocyte hypertrophy. However, it is also possible that many of the enzyme-inducing compounds in our dataset were CAR activators. This may explain why the LightGBM model indicated significant contributions only from *Cyp2b1*, a representative CAR target gene.

We conducted a comparative analysis of SHAP values for two samples, Phenobarbital/High/28-day and Nifedipine/High/28-day, which exhibited similar levels of *Cyp2b1* induction but differed in the occurrence of centrilobular hepatocyte hypertrophy (Figure 4). Since phenobarbital and nifedipine are thought to induce *Cyp2b1* through the activation of CAR and PXR, respectively [35,36], this difference in induction mechanisms may influence the development of centrilobular hepatocyte hypertrophy. However, since other compounds that activate PXR also cause centrilobular hepatocyte hypertrophy [6], the difference in *Cyp2b1* induction mechanisms was not regarded as the primary factor in the development of hepatocyte hypertrophy.

The quantification of each gene’s contribution to prediction through SHAP value analysis revealed that Nifedipine/High/28-day exhibited smaller SHAP values for genes with positive contributions, including *Cyp2b1*, compared to Phenobarbital/High/28-day. Furthermore, some DME genes, such as *Mgst2*, which showed positive contributions in Phenobarbital/High/28-day, demonstrated negative contributions in Nifedipine/High/28-day. These results suggest that while the Phenobarbital/High/28-day and Nifedipine/High/28-day samples demonstrated similar levels of CYP2B1 induction, the former showed the induction of additional DMEs, whereas the latter did not.

Given that cell size correlates with intracellular protein content [37,38,39], it is suggested that the induction of CYP2B1 alone is insufficient to cause centrilobular hepatocyte hypertrophy and that increasing intracellular protein levels through the induction of multiple DMEs is essential.

In this study, after evaluating multiple machine learning models based on various principles for predictive model construction, LightGBM demonstrated higher accuracy (including ROC-AUC) than other prediction models (Figure 1). Previous reports have also shown that LightGBM exhibits greater predictive accuracy than other machine learning models [40,41]. In particular, models based on gradient-boosting algorithms, such as LightGBM, tend to show high predictive performance with large-scale datasets [42]. The dataset used in this study was medium-sized, comprising approximately 10,000 explanatory variables and about 5000 data points, which likely explains why complex algorithms like LightGBM achieved high accuracy. Our current results are consistent with previous studies on toxicity prediction using machine learning.

We aimed to identify the genes involved in centrilobular hepatocyte hypertrophy by developing a toxicity prediction model and evaluating the toxicity mechanism based on the contribution of each feature to the predictions. This method is likely valuable for other analyses that utilize large-scale toxicogenomics databases. For example, Open TG-GATEs contains measurement values for liver injury markers, such as alanine aminotransferase, as well as information on various hepatic pathological findings, including hepatocellular necrosis. Therefore, the approach employed in this study can be used to estimate the mechanisms underlying different types of adverse effects.

This study has some limitations. First, we utilized the data available from Toxygates and Open TG-GATEs, with the test compounds selected by the project. Therefore, the chemical space may be biased. The results obtained in this study require validation with a different dataset in future studies. In addition, the reproducibility of the animal experiments and microarray analyses is unclear. Finally, another limitation relates to the development of prediction models. We employed well-established prediction models in this study, but only in limited numbers, along with the default hyperparameters for each model.

In conclusion, we developed machine learning-based prediction models for centrilobular hepatocyte hypertrophy in rats using gene expression data. To our knowledge, this is the first model with this combination of the objective and explanatory variables. Analyses of the rationale behind the predictions suggest that the induction of CYP2B1 and other DMEs is crucial for the development of centrilobular hepatocyte hypertrophy. These findings reinforce the hypothesis that centrilobular hepatocyte hypertrophy arises from DME induction. While the induction of these DMEs is considered a key factor in hepatocyte hypertrophy due to their role in increasing intracellular protein levels, further research is necessary to clarify the detailed mechanisms involved in this process.

## 4. Materials and Methods

### 4.1. Dataset

Pathological findings of the liver in rat repeated-dose toxicity studies were obtained from Open TG-GATEs [20], while gene expression data were obtained from Toxygates [21]. Open TG-GATEs provides gene expression data and toxicity evaluation results (including liver and kidney pathological findings and blood biochemistry results) from in vitro tests using rat and human primary hepatocytes, as well as in vivo tests using rats, where 170 compounds were administered at three dose levels over multiple durations. For in vivo studies, liver and kidney gene expression data were collected using the Affymetrix GeneChip system (Rat Genome 230 2.0 Array). These data were obtained in triplicate under identical dosing conditions (compound, dose, administration period). In Toxygates, the gene expression data from Open TG-GATEs was preprocessed and presented as logarithmic values with base 2 (Log2(fold-change)) relative to the vehicle control group.

Since these databases manage data using identical IDs to identify samples, representing data such as gene expression, pathological findings, and blood biochemistry results obtained from an individual rat under specific dosing conditions, we obtained liver pathological finding data from rat repeated-dose toxicity studies via Open TG-GATEs and the corresponding gene expression data from Toxygates. Then, using the IDs as keys, we combined the two datasets to construct the final dataset containing both pathological findings and gene expression data.

We obtained gene information for the 31,014 probes included in the Rat Genome 230 2.0 Array and limited our analysis to those where one probe corresponded to a single gene. In cases where multiple probes were assigned to the same gene, we randomly selected one probe to represent the gene expression level for our analysis. The final number of probes (genes) included in the study was 14,037.

The presence or absence of centrilobular hepatocyte hypertrophy was determined by whether Liver/Centrilobular/Hypertrophy appeared in the pathological findings of Open TG-GATEs. For analytical convenience, we assigned a label of 1 for positive cases and 0 for negative cases. The labeling of centrilobular hepatocyte hypertrophy for each sample is presented in Supplementary Table S1.

### 4.2. Development of Prediction Models for Centrilobular Hepatocyte Hypertrophy

We trained six models: logistic regression [43], SVM [44], RF [45], LightGBM [23], XGBoost [24], and kNN [46], using gene expression Log2FC values as explanatory variables and the presence or absence of centrilobular hepatocyte hypertrophy as the objective variable. These models were selected based on their established track record in various toxicity predictions [40,47,48,49].

All model training was conducted in a Python 3.10 environment using Scikit-learn (1.2.2) for logistic regression, SVM, RF, and kNN, along with LightGBM (4.1.0) and XGBoost (2.0.3). For initial model comparison, we used the default hyperparameters set in the libraries. For example, logistic regression employed L2 regularization (strength: 1.0) as the regularization parameter, and SVM utilized the RBF kernel. The reason for using the default parameters was that the aim of this study was to obtain mechanistic insight into chemical-induced hepatocyte hypertrophy, rather than to construct the best machine learning model to predict it. The random seed was not fixed at this stage.

After comparing the performance of all six models, LightGBM achieved the highest ROC-AUC. For this best-performing model, we implemented early stopping to further improve generalization performance by preventing overfitting and reducing computation time. Specifically, we monitored the ROC-AUC values for validation data at each learning step. If the ROC-AUC value did not improve for 100 consecutive learning steps, we saved the model from the learning step that recorded the best ROC-AUC and terminated the training. This was independently conducted for each fold in the five-fold cross-validation. The random seed was set at 42.

To evaluate the prediction accuracy of the established machine learning models, we performed five-fold cross-validation, dividing the compounds into five groups using Scikit-learn’s StratifiedGroupKFold. This method ensured minimal differences in positive rates among the folds, and that results from the same compound were not included in different splits. The assignment of splits for the five-fold cross-validation is documented in Supplementary Table S1.

### 4.3. Evaluation of Predictive Models

We performed validation by defining four of the five folds as training data and designating the remaining fold as validation data. This process was repeated five times to ensure that each fold served as validation data once. The prediction accuracy of each model was assessed by calculating the mean of the prediction accuracy metrics across the five validation folds. The evaluation metrics used were the following: (1) accuracy (the proportion of correctly predicted samples among all samples, assessing the model’s overall performance), (2) BA (the average proportion of actual positive samples correctly predicted as positive and the proportion of actual negative samples accurately predicted as negative, a robust metric even for imbalanced data), (3) precision (the ratio of actual positive samples among those predicted as positive, crucial for minimizing false positives), (4) recall (the ratio of samples predicted as positive among actual positive samples, essential for minimizing false negatives), (5) F1-score (the harmonic mean of precision and recall, which considers the balance between these two metrics), and (6) ROC-AUC (a metric unaffected by the threshold for positives in predictions). These evaluation metrics were calculated using the following equations:

$$Accuracy=\frac{\mathrm{TP}+\mathrm{TN}}{\mathrm{TP}+\mathrm{FP}+\mathrm{TN}+\mathrm{FN}},$$

$$\mathrm{BA}=\frac{1}{2}(\frac{\mathrm{TP}}{\mathrm{TP}+\mathrm{FN}}+\frac{\mathrm{TN}}{\mathrm{TN}+\mathrm{FP}}),$$

$$\mathrm{Precision}=\frac{\mathrm{TP}}{\mathrm{TP}+\mathrm{FP}},$$

$$\mathrm{Recall}=\frac{\mathrm{TP}}{\mathrm{TP}+\mathrm{FN}},$$

$$F1\text{-}\mathrm{score}=2\times \frac{\mathrm{Precision}\times \mathrm{Recall}}{\mathrm{Precision}+\mathrm{Recall}}$$

where TP, TN, FP, and FN represent true positives, true negatives, false positives, and false negatives, respectively. A sample was considered positive if the predicted probability exceeded the overall positive rate of 0.1005.

We conducted a SHAP value analysis using the model that has the highest ROC-AUC value, as positive determination criteria do not impact this metric.

### 4.4. Calculation of SHAP Values

To evaluate each gene’s contribution to the prediction of centrilobular hepatocyte hypertrophy, we employed SHAP [25]. SHAP utilizes the Shapley value concept from cooperative game theory in machine learning, allowing us to quantify and interpret how each feature (gene expression levels in this study) influences the model’s predictions. A positive SHAP value for a given gene indicates that its expression level contributes to an increase in the predicted probability of centrilobular hepatocyte hypertrophy, while a negative value contributes to a decrease. We used TreeExplainer (https://github.com/shap/shap, accessed on 11 March 2025) from the Python SHAP library (0.44.0) for SHAP value estimation, which is optimized for rapid computation with tree-structured models such as LightGBM.

In this study, we evaluated the average magnitude of each gene’s contribution to prediction by using the mean absolute SHAP value. To calculate this value, we first computed SHAP values for each gene across all samples using the five models trained in the five-fold cross-validation. We then calculated their absolute values (absolute SHAP values). Finally, we determined the mean absolute SHAP value from these absolute SHAP values across all samples and the five LightGBM models for each gene.

### 4.5. Enrichment Analysis

To perform a biological interpretation of genes with high mean absolute SHAP values, we conducted an enrichment analysis using Metascape [50]. *R. norvegicus* (rat) was selected as both the input and analysis species, and the top 100 genes ranked by mean absolute SHAP values from the LightGBM models were used as target genes for the analysis. For background genes, we employed all 14,037 genes as explanatory variables. The analysis incorporated three databases: Gene Ontology (GO) Biological Processes, Kyoto Encyclopedia of Genes and Genomes (KEGG) Pathway, and Reactome Pathway.

### 4.6. Computational Environment and Program Codes

The analysis was conducted using Google Colaboratory, where LightGBM and XGBoost were trained on a Tesla T4 GPU. The program codes are available at https://github.com/Univ-Shizuoka-Eisei/Hypertrophy-Prediction (accessed on 11 March 2025), and the dataset used in this study is available upon reasonable request.

## Figures and Tables

**Table 1:** Summary of in vivo experimental results obtained from TG-GATEs. The numbers and proportions of positive samples for centrilobular hepatocyte hypertrophy across 134 compounds, three dosing levels, and four administration durations.

Dose Level	Treatment Duration (d)	Total Samples	Positive Samples	Positive Rates (%)
High	28	393	110	28.0
	14	420	97	23.1
	7	429	67	15.6
	3	429	40	9.3
Middle	28	423	73	17.3
	14	423	44	10.4
	7	423	26	6.1
	3	423	15	3.5
Low	28	423	24	5.7
	14	423	9	2.1
	7	423	3	7.0
	3	423	0	0
All	-	5055	508	10.1

**Table 2:** Distribution of data across each fold for five-fold cross-validation. The total number of samples, the number of positive samples, the positive rates, and the number of compounds included in each fold are presented.

Fold	Total Samples	Positive Samples	Positive Rates (%)	Number of Unique Compounds
0	1004	104	10.4	28
1	1023	114	11.1	30
2	1039	108	10.4	29
3	991	79	8.0	28
4	998	103	10.3	28

## Data Availability

The program codes are available at https://github.com/Univ-Shizuoka-Eisei/Hypertrophy-Prediction (accessed on 11 March 2025) and the dataset used in this study is available upon reasonable request.

## Supplementary Materials

The following supporting information can be downloaded at: https://www.mdpi.com/article/10.3390/ijms26104886/s1.
