# Supplementary material for: Machine Learning on Toxicogenomic Data Reveals a Strong Association Between the Induction of Drug-Metabolizing Enzymes and Centrilobular Hepatocyte Hypertrophy in Rats
Source: Int J Mol Sci. 2025 May 20;26(10):4886. doi: 10.3390/ijms26104886 (PMC12112521; doi:10.3390/ijms26104886)
Supplement: Supplementary file 1 [file ijms-26-04886-s001.zip › Ikoma_SupplFig.pdf]

Figure S1

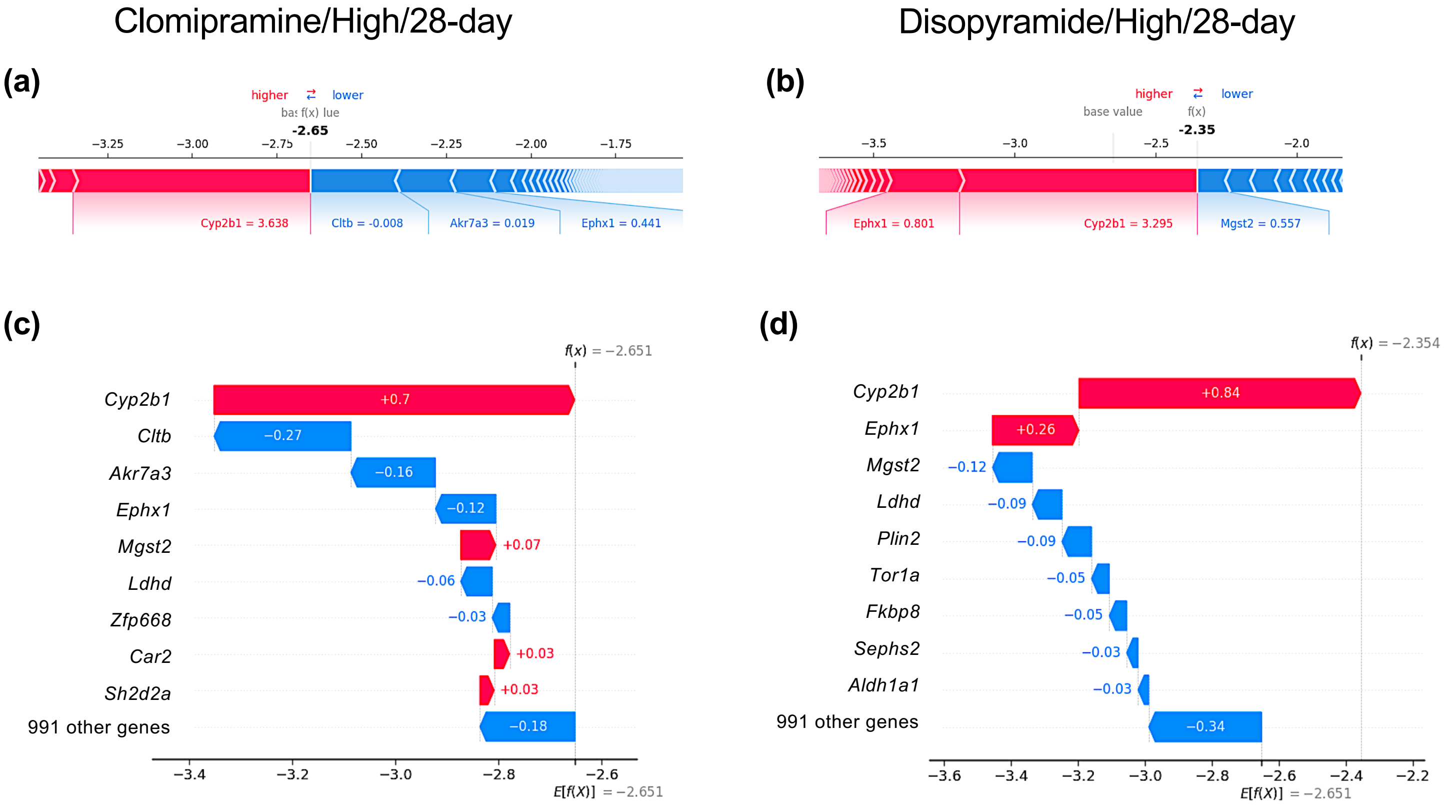

Figure S1. SHAP analysis of genes' contributions to model predictions for clomipramine disopyramide.
